# Evaluating diode laser and conventional scalpel techniques in maxillary labial frenectomy for patient perception, tissue healing, and clinical efficacy: six-month results of a randomized controlled study

**DOI:** 10.4317/medoral.26931

**Published:** 2025-02-15

**Authors:** Zeynep Tastan Eroglu, Osman Babayigit, Fatma Ucan Yarkac, Kaan Yildiz, Dilek Ozkan Sen

**Affiliations:** 1 ORCID: 0000-0002-0003-2120. Assistant professor. Necmettin Erbakan University, Dentistry Faculty, Department of Periodontology, Konya, Turkey; 2 ORCID: 000-0001-9842-6306. Assistant professor. Necmettin Erbakan University, Dentistry Faculty, Department of Periodontology, Konya, Turkey; 3 ORCID: 0000-0001-8126-585X. Associate Professor. Necmettin Erbakan University, Dentistry Faculty, Department of Periodontology, Konya, Turkey; 4 ORCID: 0009-0005-2614-4891. Research Assistant. Necmettin Erbakan University Faculty of Dentistry, Department of Periodontology, Konya, Turkey; 5 ORCID: 0000-0002-0531-1217. Assistant professor, Necmettin Erbakan University, Dentistry Faculty, Department of Periodontology, Konya, Turkey

## Abstract

**Background:**

This study aims to compare scalpel and diode laser techniques regarding patients’ perceptions, tissue healing, diastema, and periodontal clinical parameters in the treatment of abnormal labial frenum.

**Material and Methods:**

This prospective, randomized, controlled trial evaluated 43 patients (aged 18-55) requiring labial frenectomy, randomized to scalpel or diode laser therapy. Plaque index (PI) and gingival index (GI) were measured at baseline, 4 weeks, and 6 months post-surgery. Keratinized gingiva width (KGW) of maxillary central incisors and diastemas were measured at baseline and 6 months post-surgery. Postoperative pain was evaluated on days 1, 7, 14, 21, and 28 using a visual analog scale. Wound healing was assessed at 7 days and 4 weeks postoperatively, scored based on the degree of epithelialization and the presence of ulceration or necrosis.

**Results:**

At 6 months, both groups showed a significant reduction in PI, GI, and diastema (*P*<0.05). KGW increased in both groups, with a significant increase in the laser group (*P*<0.05), though baseline and 6-month KGW values were not significantly different between groups: baseline values were 5.30 ± 1.396 for the scalpel group and 5.05 ± 1.276 for the laser group, and 6-month values were 5.65 ± 1.152 for the scalpel group and 5.50 ± 1.147 for the laser group (*P*<0.05). The diode laser group had significantly lower pain scores than scalpel group on days 1, 3, and 7 (*P*<0.05). however, from day 14 onward, there was no statistically significant difference in pain scores between groups (*P*<0.05). Tissue healing was significantly faster on day 7 in the scalpel group (*P*<0.05).

**Conclusions:**

Frenectomy with diode laser effectively reduces pain, although it may delay wound healing. Laser therapy serves as a feasible alternative to the scalpel method. However, further research is necessary to fully assess its benefits and limitations in soft tissue procedures.

** Key words:**Frenectomy, diode laser, scalpel, post-operative pain, visual analog scale, wound healing.

## Introduction

The labial frenulum is a distinct fold of the mucous membrane, composed of connective tissue and muscle fibers. The periosteal insertion serves as the primary connection between the lips and the alveolar mucosa or gingiva. This anatomical feature plays a role in the stabilization and restriction of lip movements, hence mitigating the excessive exposure of gingival tissue (1). Frenal attachments in a high position might cause inadequate clearance of dental plaque and displacement of the gingival tissue due to the stress they exert (2). In such cases, it is necessary to perform a frenectomy (3).

Labial frenectomy is a surgical treatment that involves the total removal of excessive interdental tissue that is linked to the underlying bone. The goal of this procedure is to reduce tension in the marginal gingival tissues. This operation not only eradicates functional constraints but also avoids the recurrence of diastema, restores the normal anatomical structure, improves aesthetics, and aids in the prevention of periodontal diseases (4).

In the past, frenectomies were performed solely utilizing scalpels and periodontal surgical instruments. Notwithstanding, advancements in technology have presented laser surgery as a viable alternative. Periodontal surgery frequently employs several laser technologies, including carbon dioxide (CO2), neodymium-doped yttrium aluminum garnet (Nd: YAG), argon, erbium YAG (Er: YAG), and diode lasers, to perform procedures such as frenectomies and other soft tissue treatments (5,6).

Due to their advantageous properties, the use of diode lasers has seen a noTable surge in popularity in recent years. Experts often recommend their use in soft tissue treatments. Moreover, these lasers cause very little heat damage to dental hard tissues and can be used safely for making incisions in the oral mucosa and gingiva, as well as for coagulation, cleaning of pockets, and the surgical removal of periodontal cysts and pyogenic granulomas (7). Nevertheless, there is a scarcity of studies in the existing body of literature that document the clinical healing outcomes of frenectomy surgeries conducted using conventional techniques or diode lasers, particularly within short follow-up periods. In addition, there is a limited amount of research available that assesses patient comfort and the healing of tissues after surgery (8).

The aim of this study is to assess and contrast the alterations in soft tissue and the pain perceptions of patients during and after frenectomy using conventional scalpel and diode laser techniques.

Furthermore, it aims to examine the reattachment of the labial frenulum, variations in keratinized tissue following frenectomy, and changes in diastema after the procedure.

## Material and Methods

The present investigation was carried out at the Department of Periodontology, following the authorization granted by the Faculty of Dentistry Ethics Committee at Necmettin Erbakan University (Decision No: 2023/327). The clinical trial was registered on the clinicaltrials.gov database with the unique identifier NCT06548516. The Declaration of Helsinki's guidelines for the ethical conduct of experiments involving human subjects guided the conduct of the study. Before participating in the investigation, each participant executed a written informed consent form.

- Study Design

The study employed a prospective, randomized, controlled design to examine periodontal clinical parameters and patients' perception during frenectomy procedures. The primary objective was to compare the efficacy of diode laser treatment with the standard scalpel technique. The presentation of the findings adhered to the standards delineated in the Consolidated Standards of Reporting Trials (CONSORT) (9).

- Participants

Participants diagnosed with abnormal labial frenum adhesion according to the definition of Mirko *et al*. (10) were included in this study. The inclusion criteria for the participants were: 1) systemically healthy; 2) non-smoker; 3) aged between 18 and 55 years; 4) no periodontal treatment received in the last 3 months; 4) having at least 20 teeth; 5) presence of at least central incisors, lateral incisors, and canines in the maxilla; 6) not pregnant or breastfeeding; 7) no psychiatric, mental, or physical impairments; 8) diagnosed with gingival health based on the "World Workshop on the Classification of Periodontal and Peri-Implant Diseases and Conditions (11); 9) consent to participate in the study. The exclusion criteria were: 1) any systemic disease that could impede the wound healing process (e.g., diabetes mellitus and HIV infection); 2) use of antibiotics, anti-inflammatory drugs, or any other medication in the past 6 months that could affect the study's outcome; 3) any hypersensitivity reactions to paracetamol; and 4) any physical limitations or restrictions that could impede normal oral hygiene procedures.

- Sample size calculation

A power calculation was conducted to determine the required number of participants for the study. We anticipated that patients would report lower pain scores following laser surgery. The sample size calculation was based on the expected change in VAS pain scores (α = 0.05, Cohen's d = 1.64) as the primary outcome (12). With a power of 99% and α = 0.05, the minimum number of subjects required for comparison was 20 per group. To account for potential dropouts, 23 participants were included in each group.

- Study groups and randomization

**Figure 1:**
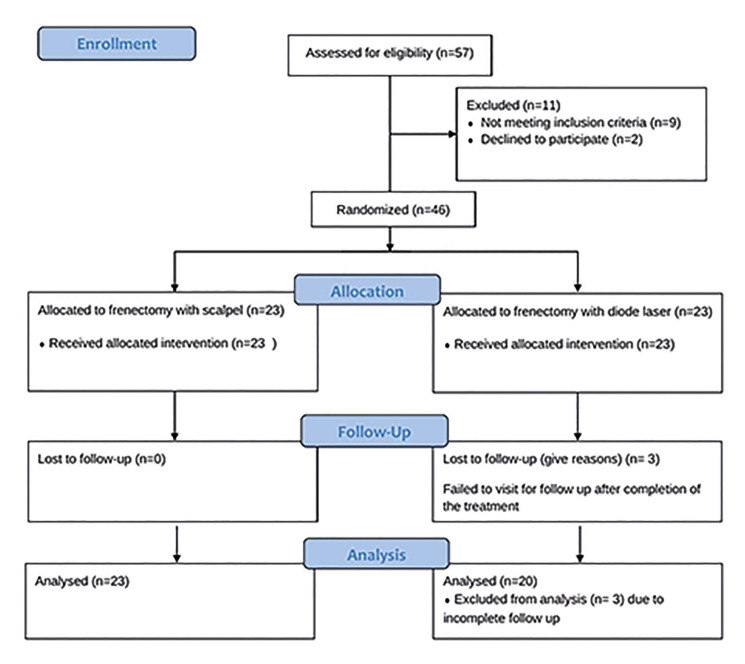
Consort flow diagram.

Fig. 1 displays the flow diagram of the investigation. A comprehensive medical and dental history evaluation, as well as intraoral and radiographic examinations, were conducted on all subjects before any surgical procedure. Of the 57 patients evaluated for eligibility, 11 were excluded: 9 did not meet the inclusion criteria—3 had a history of antibiotic use within the previous 6 months, 2 had received periodontal treatment in the last 3 months, 1 presented with diabetes, and 3 were identified as smokers during screening—and 2 declined to participate. Participants who fulfilled the inclusion criteria were given oral hygiene instructions that were customized to their requirements. Initially, the clinical periodontal parameters of all participants were measured. Then, a mechanical cleaning procedure was carried out using an ultrasonic scaler (Cavitron; Dentsply International) and hand instruments (Gracey, SG 5/6, 7/8, 11/12, 13/14; Hu-Friedy Ins. Co.).

The study enrolled a total of 46 participants and randomly assigned them to one of two groups using a computer-generated randomization method (www.randomizer.org): the experimental group, which underwent diode laser surgery (*n*=23), and the control group, which underwent conventional scalpel surgery (*n*=23). Three patients in the experimental group were excluded from the study because they failed to show up for their planned follow-up appointments.

- Treatment Procedure

Control group (conventional scalpel surgery): Following the administration of local infiltrative anesthesia using a 1:100,000 combination of articaine HCL and epinephrine, we grasped the frenum using a straight hemostat in the vestibule. The tissue adjacent to the upper and lower surfaces of the hemostat was then incised using a No. 15 scalpel. The hemostat removed the excised diamond-shaped portion of the frenum. The surgeon then used a scalpel to make a horizontal incision on the periosteum to prevent fiber reattachment. We primarily closed the wound site with absorbable sutures (4-0 vicryl, Ethicon, Ohio, USA), and removed them one week post-operatively.

Diode laser group: The frenectomy procedure was performed under local infiltrative anesthesia, specifically articaine HCL with epinephrine 1:100,000. A straight hemostat, similar to that used in the scalpel group, was employed to secure the frenum. Laser energy was applied to the upper and lower sections of the frenum adjacent to the hemostat, utilizing a diode laser device equipped with a 400 µm diameter, plain-ended optical fiber tip. Frenectomy was performed using 940-nm indium gallium arsenide phosphide (InGaAsP) semiconductor diode laser (BioLase, California, USA). The laser was operated in pulsed CP2 wave mode at the power of 1.2 watt (W).

- Post-treatment procedure

After the frenectomy procedures in all groups, patients received oral hygiene instructions and were advised to consume soft and cold food for the next 12 hours (3). Additionally, a 0.12% chlorhexidine gluconate mouth rinse was prescribed to be used once a day for one minute over five days. For pain relief, 500 mg of paracetamol (1-2 Tablets) was prescribed as needed, with patients instructed to record the dosage and frequency of use.

- Clinical evaluation

Z.T.E. performed all surgical procedures, while a calibrated single examiner (K.Y.,) performed all clinical evaluations. Patients in the scalpel group underwent primary wound healing with sutures, whereas those in the diode laser group did not receive any sutures. Consequently, due to the inherent differences in treatment protocols, blinding of both clinicians (Z.T.E., K.Y., and O.B.) and patients to group assignments was not feasible throughout the study. We conducted a calibration exercise until the agreement coefficient reached a level of 90% to ensure consistency in K.Y.'s measurements. This calibration process consisted of evaluating three patients on two separate occasions within 24 hours. The calibration was thought to work if there was less than a 3% difference between the first and second measurements of keratinized gingival width (KGW) after 24 hours (13). Plaque index (PI) and gingival index (GI), were measured using a periodontal probe from the University of North Carolina (PCPUNC15; Hu-Friedy Mfg. Co. Inc., Chicago, IL, USA). At baseline, postoperative week 4, and month 6, all periodontal measurements were taken at six sites per tooth as part of a full-mouth evaluation. KGW on maxillary central incisors teeth, the distance between the frenum's insertion and the papilla's highest point (14), interdental papilla width, and the amount of diastema (The midline diastema in the maxillary central incisors was recorded millimetres from the height of the line of the adjacent teeth or at the point of greatest convexity on the proximal surface (15)) were measured at baseline and month 6. Before the surgeries, the labial frenulum attachments were classified into four types: mucosal (insertion of frenulum to the mucogingival junction), gingival (insertion of frenulum to the attached gingiva), papillary (insertion of frenulum to the interdental papilla), and papilla-penetrating (insertion of frenulum passes right up to the papilla) (16). Pain was evaluated using the visual analog scale (VAS) on postoperative days 1, 3, 7, 14, 21, and 28. The scale was comprised of a horizontal line with values ranging from '0' to '10', where '0' indicated the absence of pain and '10' indicated the presence of severe discomfort. Tissue healing was evaluated by O.B. on the 7th day and 4th week after surgery using the following scoring system (3): 1: complete epithelialization, 2: incomplete epithelialization, 3: ulcer, and 4: tissue defect or necrosis.

- Statistical analysis

Each patient was considered a single statistical unit, and the data were analyzed using SPSS 26 (IBM Amarok, NY, USA) with a significance threshold set at *p* < 0.05. The Kolmogorov-Smirnov test was employed to assess the normality of the data distribution. Descriptive analysis of the study population was initially conducted, including the frequency distribution of categorical variables and the mean with standard deviation for quantitative variables. Categorical variables between groups were compared using the chi-square test. The Student’s t-test was used to compare group means at baseline and post-surgery, while repeated measurements of clinical parameters were analyzed using the paired-sample t-test.

## Results

A total of 46 patients were initially enrolled; however, 3 discontinued follow-up, resulting in the evaluation of 43 patients (female/male = 34/29) with a mean age of 27.4 ± 9.98 years (range 18-54). No statistically significant differences were observed between the scalpel and diode laser groups in terms of mean age, gender distribution, or frenum attachment types (*p* > 0.05; Table 1).

The clinical data, including PI, GI, and KGW of the maxillary central incisors, as well as papillary height, papillary width and diastema at baseline and 6 months postoperatively, are summarized in Table 2. No significant differences were observed between the treatment groups in terms of these clinical parameters at baseline and 6 months postoperatively (*p* > 0.05). Both groups demonstrated significant decreases in PI and GI at 6 months (*p* > 0.05). In the diode laser group, the mean KGW values of the maxillary central incisors increased significantly at 6 months postoperatively compared to baseline (*p* > 0.05), whereas no statistically significant change was observed in the scalpel group (*p* > 0.05). Both groups showed a significant increase in papillary height and a significant decrease in diastema at the 6-month follow-up compared to baseline (*p* > 0.05). The migration of the frenulum insertion extended to the mucogingival junction, and no instances of recurrence were detected in any patient during the 6-month postoperative assessments. At the 6-month, all individuals' frenulum classifications had achieved mucosal type.

**Figure 2:**
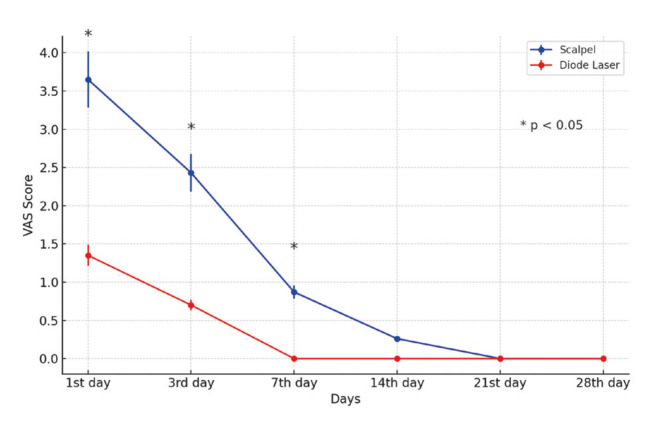
Comparison of VAS scores between groups.

A statistically significant difference in pain levels was seen between the two groups at 1 day (*P*=0.000), 3 days (*P*=0.000), and 7 days (*P*=0.000) following the surgical procedure. The diode laser group consistently reported lower score of pain. Nevertheless, the disparity observed 14 days, 21 days, and 28 days after the surgery did not reach statistical significance (*P*=0.098) (Fig. 2).

**Figure 3:**
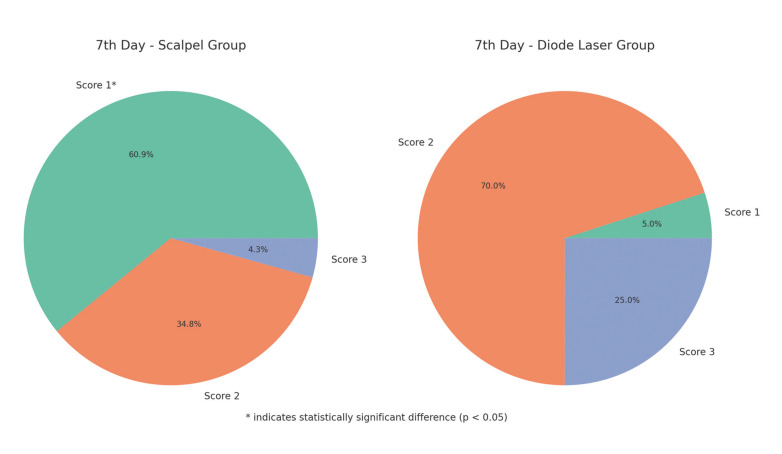
Comparison of healing scores between groups on the 7th day.

**Figure 4:**
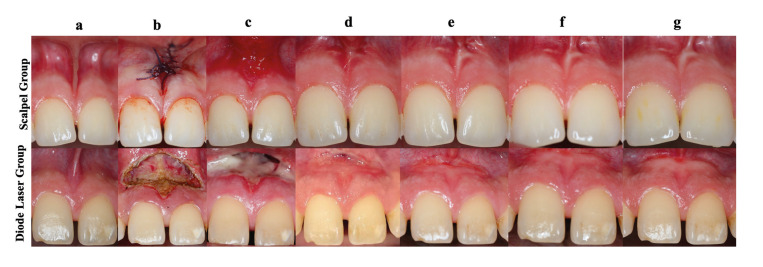
Comparison of the scalpel group and the diode laser group (a) baseline intraoral view; (b) the wound site after operation; (c) the clinical appearance of healing on post-operative 7th, (d)and 14th day, (e) 4th week, (f) 3rd and (g) 6th month.

At 7 days postoperatively, the two groups showed a significant difference in tissue healing (*P* = 0.000). The scalpel group exhibited significantly faster tissue healing than the diode laser group (*P* = 0.000) (Fig. 3). In the 4th postoperative week, all groups demonstrated completely epithelialized wound areas. Fig. 4 presents intraoral photographs that illustrate the tissue healing progression over time for both groups.

## Discussion

This randomized, prospective, and controlled study compared conventional scalpel surgery with diode laser surgery in performing a frenectomy of the upper labial frenum. To our knowledge, this study is one of only a few studies that have compared long-term clinical assessments of soft tissue and the level of subjective complaints and tissue healing after using diode laser and scalpel-assisted frenectomy techniques. The diode laser group reported significantly lower pain levels on the first, third, and seventh days post-surgery. Nevertheless, by the fourteenth day, degrees of pain were not different between groups. While all patients recovered completely by week four, the group using scalpels showed better early postoperative healing.

This study also illustrated the distribution of frenulum attachment groups in patients who required frenectomy. Mirko *et al*. (10) and Jindal *et al*. (17) observed that the mucosal type was the most prevalent, along with the gingival type as second, followed by the papillary-penetrating type and the papillary type. In our study, patients with the mucosal type of frenulum attachment were excluded from the study, as this kind of attachment does not necessitate surgical removal. The majority of participants in our study exhibited the gingival type of frenulum. Notably, there was a total lack of papilla penetrating attachment. The lack of this particular frenulum could be attributed to factors such as the size of the sample, features of the population, or random fluctuations.

Conventional frenectomy surgery often results in a large, triangular wound that requires sutures to control bleeding (6). In contrast, the diode laser-assisted approach is frequently chosen as an alternative or adjunct to traditional scalpel methods for various oral soft-tissue surgeries. The diode laser, a semiconductor device that converts electrical energy into light, is highly regarded for soft-tissue procedures because it does not interact with hard tissues (18). It is widely used in clinical practice due to its ergonomic advantages, including portability, quick setup, compact size, and low cost (19).

In the literature, while numerous studies on diode laser frenectomy exist, only a few have examined soft tissue changes post-surgery. In the study by Uraz *et al*. (6), significant gains in KGW, attached gingiva width, and thickness were observed in both laser and scalpel groups, with no significant differences between them. In our study, an increase in KGW was observed for maxillary central incisors in both groups, with the diode laser group showing a significantly greater improvement.

Microbial dental plaque accumulation and inadequate oral hygiene are major risk factors for periodontal diseases, making plaque removal essential for successful periodontal therapy. In our study, oral hygiene and gingival health were assessed using the PI and GI. Since all participants received preoperative oral hygiene instructions and nonsurgical periodontal therapy, baseline PI and GI scores were similar in both the scalpel and diode laser groups, consistent with findings by Ozener *et al*. (18). Ozener *et al*. observed a significant increase in PI and GI scores at 6 weeks postoperatively only in the scalpel group, likely due to plaque accumulation around sutures and inadequate oral hygiene due to discomfort and pain. Nevertheless, our study demonstrated a reduction in PI and GI scores in both groups at 4 weeks and 6 months following the surgery. These findings indicate that the surgery did not hinder oral hygiene behaviors. Instead, the procedure may have contributed to the formation of an anatomical change that facilitated improved oral hygiene practices.

The literature on diastema closure following frenectomy is quite limited (20). In a randomized clinical study by Tanik *et al*. (21), 50 patients aged 13-53 with frenum-induced diastema underwent conventional frenectomy and were evaluated for changes in diastema one year later, showing a significant reduction in the diastema. In our study, consistent with the findings of Tanik *et al*., a significant reduction in diastema was observed in both groups at the 6-month follow-up.

Typically, at least one month is needed for the formation of an intact mucosa at the new attachment position of the frenulum (18). In our study, we evaluated the reinsertion of frenulum attachments at 4 weeks postoperatively. Both the scalpel and diode laser groups showed successful relocation of the frenulum to the mucogingival junction by the 4th week, with no recurrence observed. At the 6-month follow-up, there was no movement in the interdental papillary region under tension, indicating a successful outcome. Similarly, Özener *et al*. (18) reported no recurrence in both groups at 6 weeks and 12 months postoperatively. However, Pie-Sanchez *et al*. (22) found that in a study of 50 pediatric patients undergoing frenectomy with either an ER: YAG or CO2 laser, the frenulum attachment migrated to the mucogingival junction 4 months postoperatively, regardless of the laser type used.

The subjectivity of pain is a significant challenge in accurately assessing its experience (23). VAS is widely utilized due to its efficacy and ease of interpretation as a short-term assessment tool for individual patients and healthcare professionals (24). In this study, pain was evaluated using the VAS on the 1st, 7th, 14th, 21st, and 45th days following the surgical procedure. Our results showed that pain scores were significantly lower in the diode laser group compared to the scalpel group on days 1 and 7 post-surgery. These results align with earlier studies that showed reduced VAS scores for laser-assisted frenectomy in comparison to traditional scalpel-assisted procedures (3,6,8). The diminished pain experienced by the diode laser group can be attributed to the procedure's lessened invasiveness, leading to a smaller surgical wound area, and the absence of sutures that could potentially amplify pain perception. Furthermore, the phenomenon of pain reduction could perhaps be attributed to the impact of the laser on nerve terminals, thereby impeding their ability to establish anastomoses (25).

The healing process varies among different tissues, influenced by their specific nature and function. In conventional frenectomy, primary wound healing is facilitated by suturing the surgical site, whereas laser-assisted frenectomy relies on secondary wound healing, where the wound is left open to heal naturally. Sezgin *et al*. (8) reported no significant difference in the epithelization period between conventional frenectomy with primary closure and laser-assisted frenectomy with secondary healing at both 7 and 14 days. Conversely, Fisher *et al*. (26) found that laser-induced wounds demonstrated faster recovery and reduced scar formation compared to conventional methods. In a study by Sobouti *et al*. (3), tissue healing was significantly more advanced in the 980 nm laser group than in the scalpel group at 7 days postoperatively, though no significant differences were observed at later time points. In our study, the scalpel group showed superior wound healing at 7 days postoperatively; however, complete healing was observed in all groups by the 30th day. The observed delay in wound healing following laser treatment may be due to thermal damage incurred during the procedure and the fact that the wound area is left to secondary healing in laser-assisted frenectomy (8).

This study had certain limitations. Blinding of participants was not achievable due to the presence of sutures in the wound site until the 2nd week, which may have introduced bias. Additionally, it would be beneficial to compare the diode laser with other lasers, such as CO2 and Nd: YAG, in future studies focused on oral soft tissue surgical procedures. Postoperatively, we prescribed chlorhexidine mouthwash, which is considered the gold standard for oral antiseptic treatment. However, recent research has associated chlorhexidine with potential side effects, including delayed healing (27). Therefore, future studies should investigate alternative topical treatments. Another limitation is that patients' daily stress levels and psychological states could have influenced their pain perceptions, potentially affecting the study's findings. Furthermore, the menstrual cycle may play a significant role in pain perception among female patients, and this factor should be accounted for in future research (28). Additionally, it would be beneficial to compare the diode laser with other lasers, such as CO2 and Nd: YAG, in future studies focused on oral soft tissue surgical procedures.

In conclusion, laser therapy can be incorporated as an alternative or complementary approach to traditional methods. Within the limitations of this study, it is concluded that both conventional scalpel-assisted and diode laser-assisted frenectomy are effective in maintaining the new position of the frenulum across various abnormal placements. Clinical findings indicate that diode lasers provide better patient outcomes, particularly in terms of reduced pain, when compared to the scalpel method. Despite some drawbacks, such as lateral heat damage, delayed wound healing, the need for operator skill, and higher costs, the diode laser remains a dependable option for soft tissue surgeries like frenectomy due to its effectiveness, safety, and reduced patient pain perception. Nevertheless, further longitudinal studies with larger sample sizes are required to conclusively determine the superiority of laser techniques over traditional scalpel methods in frenectomy procedures.

## Figures and Tables

**Table 1:** Demographic and Clinical Characteristics of Patients in the Scalpel and Diode Laser Groups.

Characteristics		Conventional group	Diode laser group	*p*
Number of the patient *(%)*		23 (53)	20 (47)	-
Age *(Mean ± SD)*		26.96±9.33	27.9±10.9	0.761^‡^
Gender, N *(%)*	Male	3 (13)	6 (30)	0.263^?^
	Female	20 (87)	14 (70)	
Type of frenum attachment, N *(%)*	Class II	20 (87)	18 (90)	0.569^?^
	Class III	3 (13)	2 (10)	

^?^ Chi-square test, *p* < 0.05; ^‡^ Student's t-test, *p* < 0.05.

**Table 2:** Groups Comparisons of Clinical Parameters at Baseline, Postoperative 4 Week and Postoperative 6 Months.

Clinical Parameters		Conventional group Mean ± SD	Diode laser group Mean ± SD	*p* ^‡^
PI (0 to 3)	Baseline	1.87±.570	1.90±.400	0.825
	4 Weeks	1.44±.275	1.50±.196	0.412
	6 months	1.37±.386	1.51±.334	0.227
	*p* ^?^	0.000	0.002	-
GI (0 to 3)	Baseline	1.74±.326	1.71±.250	0.770
	4 Weeks	1.39±.154	1.43±.162	0.456
	6 Months	1.21±.358	1.19±.257	0.860
	*p* ^?^	0.000	0.000	-
Keratinized gingiva (11), mm	Baseline	5.76±1.445	5.05±.999	0.072
	6 Months	6.11±1.331	5.75±.851	0.307
	*p* ^?^	0.133	0.002	-
Keratinized gingiva (21), mm	Baseline	5.30±1.396	5.05±1.276	0.539
	6 Months	5.65±1.152	5.50±1.147	0.667
	*p* ^?^	0.088	0.046	-
Papillary height, mm	Baseline	3.57±1.300	3.95±1.468	0.367
	6 Months	7.87±1.842	8.60±1.429	0.158
	*p* ^?^	0.000	0.000	-
Papillary width, mm	Baseline	3.37±.742	3.50±.946	0.615
	6 Months	3.46±.752	3.53±.966	0.795
	*p* ^?^	0.400	0.189	-
Diastema	Baseline	1.52± 0.58	1.5± 0.74	1.0
	6 Months	1.35± 0.56	1.23± 0.58	0.577
	*p* ^?^	0.043	0.045	-

^?^Paired-sample t-test, *p* < 0.05, Compared the baseline measurements to those taken at 6 months; ^‡ ^Student's t-test, *p* < 0.05, Compared to the group.

## Data Availability

The data that support the findings of this study are available from the Correspondence, [ZTE], upon reasonable request. Written and verbal consent was acquired from all participants who agreed to participate in the study.
